# Psychological Preparation for Cardiac Surgery

**DOI:** 10.1007/s11886-020-01424-9

**Published:** 2020-10-10

**Authors:** Stefan Salzmann, Miriam Salzmann-Djufri, Marcel Wilhelm, Frank Euteneuer

**Affiliations:** 1 Division of Clinical Psychology and Psychotherapy, Philipps University of Marburg, Gutenbergstraße 18, 35032 Marburg, Germany; 2 Department of Cardiovascular Surgery, University Hospital Giessen, Giessen, Germany; 3 Department of Psychology, Clinical Psychology and Psychotherapy, Medical School Berlin, Berlin, Germany

**Keywords:** Psychological preparation, Intervention, Psychosocial risk factors, Expectation, Psychosocial support, Cardiac surgery

## Abstract

**Purpose of Review:**

To review the current state of preoperative psychological preparation to improve outcomes after cardiac surgery.

**Recent Findings:**

Preoperative psychosocial factors are associated with short- and long-term outcomes after cardiac surgery. There are several approaches to optimize patients’ preoperative psychological status with promising effects on postoperative outcomes (e.g., less complications, improved quality of life). Preoperative psychological preparation often aims to improve patients’ knowledge or social support and to modify and optimize expectations and illness beliefs.

**Summary:**

Preoperative psychological preparation is gaining importance for cardiac surgery. However, patients’ psychological status still does not get as much attention as it deserves. Preoperative psychological preparation seems to have positive effects on postoperative outcomes. Since overall evidence is still weak, further studies are warranted to understand which intervention works best for whom and why.

## Introduction

Cardiovascular disease is one of the most common causes of restricted quality of life, disability, and mortality, with high costs for the healthcare system [1, 2]. Cardiac surgeries aim to reduce disability, physical symptoms, and morbidity and improve quality of life [3]. However, despite an ongoing progress of science and technology to improve surgical outcomes, a substantial amount of patients has problems to recover psychologically and physically from cardiac surgery [4–6]. Cardiac surgery is a stressful life event associated with physical and psychological impairments such as anxiety, fear, depression, and pain [7]. While former efforts have primarily focused on surgical and anesthetic techniques, somatic comorbidities, and diet and physical activity, growing evidence suggests the importance of psychological preparation to improve postoperative surgical outcomes [8, 9], even for very invasive procedures such as cardiac surgery.

Several well-documented psychosocial risk factors for cardiovascular disease [10•, 11, 12] are also predictors for outcomes after cardiac surgery. These factors may involve demographic variables (e.g., age and/or gender) [13, 14], depressive symptoms [4, 15–18], anxiety [15, 16, 19–21], chronic (work and/or family) stress [11, 22], socioeconomic status [11, 14, 16, 17, 23], social support [18], health behaviors [24–26], marital status [18], and preoperative expectations [27–29] and illness beliefs [30]. Unfortunately, this knowledge is not sufficiently considered by current guidelines and daily routines in cardiac surgery [31••]. For instance, risk scores such as the EuroSCORE do not consider psychological variables [32].

**Fig. 1:**
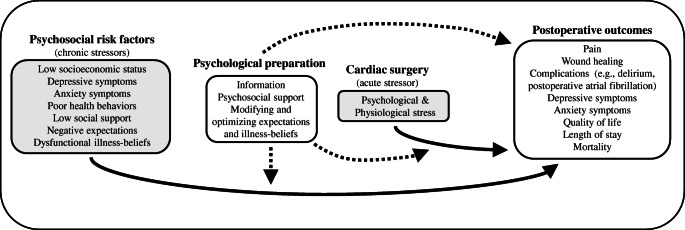
The PSY-PREP-model (psychological preparation before cardiac surgery) as a heuristic model which summarizes potential effects of preoperative psychosocial risk factors and acute stress due to cardiac surgery on postoperative outcomes (solid arrows). The model further displays that preoperative psychological preparation not only aims to modify these effects but also directly targets postoperative outcomes (dashed arrows)

The rational of preoperative psychological preparation is to modify one or more of these chronic stress factors and to help patients cope with the acute stress of the cardiac surgery to improve postoperative outcomes. Figure 1 illustrates a heuristic model for preoperative psychological preparation (i.e., PSY-PREP model). Postoperative outcomes do not only include “classical surgery outcomes” such as mortality, complications, length of stay (LOS) in the hospital, and healthcare costs but also patient reported outcomes such as pain, quality of life, anxiety, depressive symptoms, or satisfaction with the medical treatment. While interventions of rehabilitation aim to improve postoperative outcomes, preoperative psychological preparation can be considered “prehabilitation,” a term that captures approaches to optimize surgical outcomes by means of preoperative approaches [26]. With respect to cardiac surgeries, however, there is a lack of evidence-based interventions for psychological preparation before undergoing surgery. A variety of mechanisms may explain why psychological preparation could influence the process of surgical recovery: Cognitions and emotions influence behavior (e.g., physical activity, adherence to medication, compliance) and may thus be relevant for rehabilitation. Emotions such as anger or sadness can increase pain sensations [33]. Perceived stress is associated with psychoneuroimmunological mechanisms which may delay wound healing and increase sickness behavior [34–36]. Psychological interventions which influence these psychological factors may thus improve postoperative outcomes [37]. In this article, we review recent developments in psychological preparation for patients undergoing cardiac surgery.

## Interventions for Psychological Preparation Before Cardiac Surgery

The term “psychological preparation” is not clearly defined. From our perspective, it includes a variety of techniques to alter cognitions, emotions, or behaviors which may improve (e.g., expectations, perceived social support) or reduce (e.g., depressive symptoms, anxiety, perceived stress) the probability for optimal recovery. In an early review, Johnston and Vogele (1993) described the following types of preoperative psychological interventions being beneficial for surgical patients [38]: providing procedural information (information on the process describing what, when and how something will happen), sensory information (describing what it will feel like), behavioral instruction (information about what a patient should do such as when a patient should return to usual activities), cognitive intervention (aiming to alter how a patient thinks about surgery; it may include developing a different perspective and distraction), relaxation techniques (systematic instructions for physical and cognitive strategies to increase relaxation and feelings of being calm), hypnosis and emotion-focused interventions (aiming to enable patients to regulate and manage emotions such as understanding and accepting emotions). Most interventions comprise a mixture of these types. Table 1 summarizes potential elements of psychological preparation before surgery.

**Table 1:** Overview of elements of interventions for psychological preparation before cardiac surgery

Element	Aim/examples
Information/education	
Assessing what the patient already knows	To enable the clinician to tailor the required information individually
Providing the patient with procedural information (what will happen), sensory information (what will it feel like), and behavioral instruction (what the patient should do)	Reducing the patient’s anxiety and uncertainty, e.g., information about the ICU stay and first steps after surgery
Psychosocial support	
Encouraging the patient to use resources	For example, spouse, family, or friends and preferred coping strategies
Asking how the patient thinks and feels about the surgery, what his worries or anxieties are, or what he wants to talk about	To improve the patient’s emotional condition and to find out about a patient’s preferred coping style (information seeking vs. avoidance)
Listening, validating, and normalizing patient’s feelings and thoughts	To show and signal that the patient’s thoughts and feelings are adequate and part of a normal reaction considering the circumstances
Helping the patient to express (negative) emotions or feelings	Fostering emotional expression to reduce patient’s emotional burden
Respecting if a patient does not want to talk about specific topics	Respecting the patient’s autonomy and individual coping style
Optimizing expectations and illness beliefs	
Assessing patient’s illness concept and expectations	To be able to identify whether or not the patient has an adequate and functional concept of his heart disease and positive but realistic expectations
Creating an “action plan” of when, how, and what activities the patient will be able to return to	To help the patient to concretely plan when s/he will be able to return to important activities (s/he might not be able to perform shortly before surgery due to the cardiac disease)
Increasing personal control expectations and coping strategies	Collecting coping strategies with the patient for side effects of the surgery to reduce the patient’s distress in the ICU; e.g., when the patient experiences pain, the patient may ask for analgesic medication or use distraction or relaxation techniques; showing that the patient can influence his future health by considering health behaviors and discussing (changeable) risk factors such as smoking, diet, and exercise to prevent another surgery in the future
Increasing treatment-related control and outcome expectations	To foster the patients’ positive expectations that the surgery will help to improve his/her cardiac disease (e.g., by discussing probable outcomes, experience and confidence of the surgeons, and other healthcare professionals); promote cooperation between the patient and the medical team; increase trust
Imagination exercise using a positive image for the time after surgery	To help the patient relax, experience positive emotions when thinking about his individualized positive, but realistic, future

### Preoperative Education

Lack of information and uncertainty are associated with preoperative anxiety [37]. Increased preoperative anxiety has been associated with postoperative complications such as atrial fibrillation, myocardial infarction, higher rates of readmissions, increased healthcare utilization and higher mortality rates in coronary artery bypass graft (CABG) patients [15, 16, 19–21]. An approach to lower preoperative anxiety is thus to provide patients with preoperative information or education. Preoperative education involves providing patients with relevant information (e.g., as a booklet, video, audiotape, or discussion) about the surgery and the postoperative time frame [39]. Healthcare professionals try to help patients to gain a better understanding of the surgical procedure which may minimize worries, anxiety, and uncertainty. Content of education involves information about expected experiences (e.g., anxiety), expected sensations (e.g., pain), and probable outcomes in individualized or group sessions. This may help to reduce the discrepancy between expected and experienced sensations or events. For instance, knowing that experiencing discomfort is part of the normal surgical experience and not an indicator that something went wrong might help patients to cope with it. A systematic review and meta-analysis indicated that preoperative education reduced anxiety among patients undergoing CABG surgery, but not pain, depressive symptoms, and length of hospital stay [39]. In one very recent study, a preoperative individualized education intervention reduced the incidence of postoperative delirium in cardiac surgery patients [40]. A 40-min intervention in which patients had the opportunity to visit an unoccupied operating room and the intensive care unit and met with staff and other inpatients (“orientation tour”) led to reduced preoperative anxiety in candidates for CABG surgery compared with that in a control group [41]. An individualized approach seems to be crucial to meet the patient’s needs. For instance, too much information may also increase anxiety in patients who tend avoid threats cognitively instead of overcoming their anxiety with the help of additional information [42]. More trials seem to be necessary to come to sound conclusions regarding the overall effectiveness of preoperative education for patients planned to undergo cardiac surgery [39]. Furthermore, most studies have been conducted in Western countries and it is unknown whether findings can be translated to non-Western contexts [43].

### Providing Psychosocial Support

Psychological or social support is frequently provided in addition to individualized information. Previous studies indicated patients’ desire for psychosocial support before undergoing CABG surgery [44, 45]. In a recent study, a brief (30 min) nurse-delivered intervention involving individualized information and emotional support 1 day before surgery reduced CABG patients’ preoperative and postoperative anxiety compared with standard medical care (SMC) only [46•]. Time spent on the intensive care unit (ICU) and in-hospital mortality was not different from a SMC group. The intervention focused on patients’ specific needs and fears and the authors concluded that healthcare professionals should be trained to better provide patients with emotional support before undergoing cardiac surgery to reduce preoperative anxiety. In another RCT, a nurse-delivered “supportive educational” intervention (comprising procedural information regarding the surgery, encouraging patients to discuss anxiety, fear and its causes, and training of relaxation techniques such as deep breathing) reduced patients’ anxiety and improved patients sleep quality before undergoing CABG surgery [47]. A supportive provider-patient interaction is considered an important and powerful mechanism with substantial influence on treatment outcomes [48]. Especially when a provider’s behavior can be described as acting warm, competent, and attentive, this supportive patient-practitioner relationship may influence treatment outcomes in a clinically significant way [49, 50].

### Modifying and Optimizing Illness Beliefs and Preoperative Expectations

Patients’ preoperative expectations and illness beliefs seem to play a crucial role for postoperative short-term and long-term outcomes. Positive preoperative expectations predict better quality of life, less disability, and less depression after CABG surgery—independent of medical risk factors [30]. There is meta-analytic evidence for the association between expectations before surgery and postoperative quality of life—irrespective of the type of surgery and disease severity [27]. Higher preoperative optimism—a generalized global expectation—is associated with lower pain intensity and physical symptoms 6–8 weeks after CABG surgery [51]. Higher optimism is also associated with lower rehospitalization rates after CABG surgery [52], as well as long-term survival rates in cardiac patients [28]. Bingel et al. (2011) found that positive treatment expectations on drug efficacy affect perceived pain (doubling of analgesic benefits of remifentanil), whereas negative expectations abolished remifentanil analgesia [53]. These effects (expectations and pain perception) were associated with objective changes in brain regions associated with coding of pain intensity [10•, 54].

Expectations related to perceived health-related personal control seem to be associated with certain key factors of patients’ short-term status after CABG surgery: In one study, higher levels of preoperative perceived personal control predicted postoperative quality of life and lower levels of depression, but not health behavior (e.g., adherence to medication or physical activity) [55]. In a cross-sectional study, heart transplant recipients with higher perceived control had lower depression and anxiety and higher quality of life ratings [56]. The potential moderating role of perceived control may be important when providing patients with information, since information without accounting for control expectations may lead to harmful effects in some situations (such as increased anxiety) [57].

In a study by Furze et al., a brief preoperative cognitive-behavioral intervention reduced dysfunctional illness-beliefs about heart disease, improved physical function and depressive symptoms before CABG surgery in comparison with SMC [58]. The intervention comprised providing patients with information on cardiac myths and misconceptions as well as procedural information, discussing dysfunctional assumptions, relaxation training, and setting of patient-centered and achievable goals for reducing risk factors (such as increasing activity levels). The study also indicated positive effects in terms of cost efficacy. Preoperative interventions for postoperative depressive symptoms might be highly valuable: There is strong evidence that patients with preoperative existing depressive symptoms have an increased risk of major adverse cardiac events, longer hospital LOS, higher levels of medical complications, an increased likelihood of lower quality of life and all-cause long-term mortality after undergoing CABG surgery compared with those patients without preoperative depressive symptoms [4, 5, 59•, 60, 61].

In one of our studies, the PSY-HEART trial, a cognitive-behavioral intervention to optimize preoperative expectations (EXPECT), improved long-term outcomes such as illness-related disability after CABG surgery compared with standard medical care (SMC). The EXPECT intervention also increased quality of life compared with the SMC and an active control intervention focusing on emotional support (SUPPORT) [62•]. EXPECT focused on the following elements: optimizing patients’ outcome expectations with regard to the advantages of the surgery, achieving a better understanding of one’s disease by correcting false assumptions, planning when patients will be able to return to positive activities, influencing controllable risk factors (e.g., smoking, diet or exercise), and collecting coping options for handling side effects of the surgery (such as asking for pain medication when being in pain). Patients were further encouraged to imagine a positive image of the time after surgery to build up positive outcome expectations about the long-term consequences of the surgery [63]. The intervention focusing on expectations significantly improved patients’ personal control expectations before cardiac surgery, reduced stress-associated biomarkers (i.e., adrenaline) after surgery, and resulted in lower inflammation after surgery (i.e., interleukin-8) and 6 months after surgery (i.e., interleukin-6) compared with the SMC [62•, 64]. Furthermore, both preoperative psychological interventions reduced the days spent in the hospital compared with the SMC indicating a positive cost to benefit ratio for brief psychological interventions prior to cardiac surgery [65]. It is likely that the positive effects of the EXPECT intervention are not only due to increased positive expectations but also due to increased positive expectations in interaction with the supportive interaction style. Both (expectation and good interaction) have been identified as powerful mechanisms of the placebo effect [48, 54]; however, it is unclear to what extent the specific components of the intervention were efficacious, or whether the “whole package” of information, providing social support and optimizing expectations is needed. Due to the typical limitations of monocenter trials, the PSY-HEART II trial started in 2019 aiming to examine whether the promising findings from the predecessor study can be corroborated by a multi-center study involving 8 locations in Germany.

## Further Evidence for Effects of Psychological Interventions in Cardiac Patients

A review examining the effects of guided imagery on physiological and psychological outcomes of ICU patients suggested positive effects on pain, anxiety, LOS, and possibly sleep quality, patient satisfaction, and cost of care [66]. However, the included studies showed a large heterogeneity, which should be considered when interpreting these results.

A Cochrane review by Ziehm et al. (2017) indicated that psychological interventions reduce mental distress in patients undergoing cardiac surgery [67]. Additionally, in a Cochrane review by Powell et al. (2016) on psychological preparation targeting adults under general anesthesia improved postoperative pain, LOS, negative affect, and behavioral recovery [37]. Both reviews considered it unlikely that preoperative preparation is associated with harmful effects; however, interventions considered in these reviews were not limited to the preoperative period only (Ziehm et al.) or to cardiac surgeries only (Powell et al.). Due to the large variation in psychological techniques, types of surgeries, and outcomes, the overall quality of evidence for the effectiveness of psychological interventions was considered low.

## Challenges in the Daily Routine of Cardiac Surgery and Clinical Implications

In the enhanced recovery after surgery (ERAS) programs, patient education is one important aspect in the perioperative time frame, although it focuses on the perioperative and the postoperative periods and not primarily on the preoperative time frame. Current guidelines suggest to provide patients planned to undergo cardiac surgery with adequate and enough information and to allow enough time for an informed and “shared” decision [31••]. However, this may be in contrast to the fact that cardiac surgery frequently includes urgent surgeries requiring rapid action without much time for the patient’s psychological preparation. Not all healthcare professionals (such as surgeons) usually receive formal training regarding communicative techniques and might have dysfunctional expectations on how to apply psychologically beneficial approaches. Resource constraints may further pose barriers for the implementation of psychological preparation in cardiac surgery settings. However, studies such as the PSY-HEART trial have already proven that preoperative psychological interventions can be integrated into hospital routine processes (in case of this RCT with additional psychologists not part of clinic personnel). But these interventions are rather easy to learn and compact training sessions could be delivered to several professionals who interact with patients (e.g., nurses, physicians, clinical social workers, psychologists).

Another challenge may be that time before undergoing the surgical procedure is usually very short (hospital admission is often 1 day before surgery). Longer time intervals (e.g., several days) in the hospital before surgery or a preparation at the cardiologist right after the decision for surgery may be helpful to provide psychological preparation. However, a long waiting period of 3 months for surgical revascularization was associated with a risk of 1 death among 80 patients in a meta-analysis [68]. There may be an optimal time frame for psychological preparation before cardiac surgery while balancing benefits and risks associated with a longer waiting period. Another challenge of delivering preoperative psychological preparation to patients at a cardiac surgery center is that a lot of patients live far from the clinic where the surgery will be conducted. Using the phone or the Internet to provide patients with psychological preparation before cardiac surgery may be part of a solution for that challenge. While phone-based approaches have been successfully used [25], there is a promising recent study indicating that a nurse-delivered Internet cognitive behavior therapy (iCBT) intervention can reduce depressive symptoms and improve quality of life in patients with cardiovascular disease [69]; however, whether such an approach may be applicable to patients before cardiac surgery is unknown.

Prehabilitation has focused on improving physical exercise as a beneficial preparation before cardiac surgery, although physical training before cardiac surgery may be difficult in patients with seriously impaired physical and especially poor cardiac function with high risk for myocardial infarction.

In the current guidelines on revascularization, a multidisciplinary heart team is suggested to decide about the best treatment option and care for each patient without mentioning psychological care [31••]. However, mental health professionals are regularly consulted in specific related fields such as heart transplantation, left ventricular assist device (LVAD) implantation, or pediatric cardiac surgery. It might be important to consider that the surgeon’s view of a successful surgery (such as complete revascularization) might substantially differ from the patient’s view of an optimal surgical outcome. Recent findings strengthen the role of psychological care in the preoperative assessment of cardiac surgery patients. To further improve patients’ preoperative care, it might not be necessary to hire additional staff, but rather train healthcare professionals who already interact with patients to provide emotional support and psychological care. Brief trainings of 1–2 days and an ongoing supervision with an experienced healthcare professional might be sufficient to enable nurses to apply new approaches such as optimizing expectations and correcting dysfunctional beliefs combined with psychosocial support to the patients they care for. Considering the already very high time pressure and increasing trend for more efficiency in the healthcare system, this approach might not be feasible without changes in current processes.

In sum, to improve the quality of preoperative care in patients undergoing cardiac surgery, we recommend the following clinical implications:

- Implementing preoperative assessments of anxiety and depressive symptoms and other psychosocial risk factors in routine clinical care. For patients with cardiovascular disease, there are existing recommendations for questions for the assessment of psychosocial risk factors and relevant screening questionnaires that may also be useful for patients planned to undergo cardiac surgery [10•, 11, 12].
- Incorporating psychosocial risk factors into risk prediction models before undergoing cardiac surgery.
- Including professionals for mental health in the heart team for decision-making regarding the best treatment option and care for each patient.
- Nurses and other healthcare professionals should be trained to provide patients with psychosocial support and other psychological interventions (e.g., optimizing expectations, correcting dysfunctional illness beliefs).

## Future Research

Despite promising findings for the effectiveness of psychological preoperative interventions for patients planned to undergo cardiac surgery, this might not be sufficient to provide every patient with the best treatment available. Most studies focusing on optimizing patients’ preoperative status focused on elective (non-emergency) surgeries. It is therefore unknown whether the promising results may be also applicable to high-risk or more complex patients. The role of moderators for treatment effects might also be very important: A recent study comparing psychological profiles between CABG surgery and valve replacement patients indicated similar anxiety and depression scores [70]. This result suggests that both groups might benefit from a similar preoperative psychological preparation to reduce anxiety and depression scores; however, most studies have been focusing on CABG surgery, while examining other surgery types might lead to important differences regarding the needs patients have and the effectiveness of specific interventions.

Furthermore, there remains considerable uncertainty regarding the causal mechanisms linking psychological morbidity to worse and psychological preparation to better clinical outcomes in cardiac disease. Who might benefit the most from which intervention and why is still unclear. Most studies so far have used interventions with multiple components often using techniques borrowed from cognitive behavior therapy (CBT). This seems logical, since no single mechanism might fully explain the complex relationship between preoperative psychological status and postoperative outcomes. However, this practice makes it hard to assess which component was relevant and which was not—even in successful preoperative interventions. Future studies might thus not only test moderators but also focus on mediators of treatment effects to analyze the essential “ingredients” of beneficial psychological preparation. A recent meta-analysis suggests that psychosocial interventions may improve immune function [71]. More research is necessary to better understand the effects of preoperative preparation on postoperative biomarkers.

All reviews mentioned so far seem to agree on one important aspect: psychological preparation before undergoing cardiac surgery seems to have positive effects on postoperative outcomes at least under some conditions. However, quality of evidence is overall low due to a low amount of studies and a large variance regarding outcomes. These few studies often show quality issues. Future high-quality studies are desperately needed to show which intervention works best for whom and why. Furthermore, more studies showing that psychological preparation can reduce costs in the long run despite producing some additional costs in the first place (e.g., additional staff to hire) and improve patients’ satisfaction might convince decision- and policy-makers to implement psychological preoperative preparation into routine clinical care. Since there seems to be more attention and experience with the impact of psychological status on cardiovascular disease in general [10•, 11, 12] and other medical disciplines (e.g., oncology), approaches could be adopted from these sources to test and further adapt them for cardiac surgery patients.

From our perspective, the principles described in this review can largely be applied to other types of surgery; however, some aspects might require special consideration in cardiac surgery. Cardiac surgery might be special compared with other surgical procedures, because the heart is a vital organ and the idea of stopping the heart on purpose (like in on-pump cardiac surgery) is usually associated with death. Furthermore, when patients are scheduled to undergo cardiac surgery, they are often confronted with questions of guilt and whether they could have prevented the surgery by living a “healthier” life.

## Conclusions

There is evidence that preoperative psychosocial factors have an impact on short- and long-term outcomes for cardiovascular disease in general and specifically for outcomes after cardiac surgery. There is further evidence that preoperative psychological preparation may improve patients’ postoperative outcomes after undergoing cardiac surgery. However, more high-quality studies are necessary to derive sound conclusions as to what kind of intervention is beneficial for whom, under what conditions, and why. Psychosocial aspects in cardiac surgery are to date not optimally considered in current guidelines, in risk prediction models, and in daily routines [16, 31••, 32]. Psychological preparation before cardiac surgery should play a more important role to improve postoperative long-term outcomes. To conclude, it seems to be important to treat not only the heart but also the mind as well to improve clinical outcomes after cardiac surgery.
